# Use and Perception of Digital Health Technologies by Surgical Patients in Germany in the Pre–COVID-19 Era: Survey Study

**DOI:** 10.2196/33985

**Published:** 2022-05-20

**Authors:** Sandra Korn, Maximilian David Böttcher, Theresa Sophie Busse, Sven Kernebeck, Michael Breucha, Jan Ehlers, Christoph Kahlert, Jürgen Weitz, Ulrich Bork

**Affiliations:** 1 Department of Gastrointestinal, Thoracic and Vascular Surgery University Hospital Carl Gustav Carus Technische Universität Dresden Dresden Germany; 2 Didactics and Educational Research in Health Science Faculty of Health Witten/Herdecke University Witten Germany; 3 National Center for Tumor Diseases (NCT/UCC) Dresden German Cancer Research Center (DKFZ) Helmholtz-Zentrum Dresden - Rossendorf (HZDR) Dresden Germany

**Keywords:** digital equity, eHealth, electronic health, mobile health, health apps, mobile health apps, eHealth literacy, mobile phone

## Abstract

**Background:**

This survey study investigates surgical patients’ use and perception of digital health technologies in Germany in the pre–COVID-19 era.

**Objective:**

The objective of this study was to relate surgical patients’ characteristics to the use and perception of several digital health technologies.

**Methods:**

In this single-center, cross-sectional survey study in the outpatient department of a university hospital in Germany, 406 patients completed a questionnaire with the following three domains: general information and use of the internet, smartphones, and general digital health aspects. Analyses were stratified by age group and highest education level achieved.

**Results:**

We found significant age-based differences in most of the evaluated aspects. Younger patients were more open to using new technologies in private and medical settings but had more security concerns. Although searching for information on illnesses on the web was common, the overall acceptance of and trust in web-based consultations were rather low, with <50% of patients in each age group reporting acceptance and trust. More people with academic qualifications than without academic qualifications searched for information on the web before visiting physicians (73/121, 60.3% and 100/240, 41.7%, respectively). Patients with academic degrees were also more engaged in health-related information and communication technology use.

**Conclusions:**

These results support the need for eHealth literacy, health literacy, and available digital devices and internet access to support the active, meaningful use of information and communication technologies in health care. Uncertainties and a lack of knowledge exist, especially regarding telemedicine and the use of medical and health apps. This is especially pronounced among older patients and patients with a low education status.

## Introduction

### Background

Information and communication technologies (ICTs) have changed our private and professional lives and are also increasingly being used in the health care sector [1,2]. ICTs cover a wide range of technologies that are often also subsumed under the term *eHealth*. da Fonseca et al [3] conducted a systematic literature review to clarify the content of the term *eHealth*. They formed 4 categories by classifying 446 publications with possible overlaps in content (mobile health [mHealth], telemedicine and telehealth, technology, and other), which we will adopt as the basic structure in this paper.

According to da Fonseca et al [3], mHealth includes the use of smartphone apps to identify, treat, and support disease. The scheduling of health examinations, use of wireless sensors to monitor patients, and transfer of these data between settings also fall under this category.

Under telehealth, the authors include interactions between providers and patients via digital means. Although the use of telehealth is highly dependent on the supply of health care professionals, it is difficult to assess the acceptance and engagement of patients receiving care in different settings. Therefore, we did not focus on this in the survey.

The technology category by da Fonseca et al [3] covers the encryption of patient medical data to protect them when they are accessed on the web. Data protection in health care facilities, the creation of system support, and the development of devices to implement digital applications also fall under this category. According to da Fonseca et al [3], the use of the Internet of Things, cloud storage, and big data can also be assigned to this category. This area is also very complex on the one hand and controlled by institutions on the other and, therefore, is not the focus of the survey.

The authors use the category *Other* to describe a combination of previous practices in new areas and a focus on costs.

To focus especially on technologies that patients can use independently and intrinsically, our survey focuses on the category mHealth, covering fitness devices and mobile apps as well as internet use and general digital health aspects. Fitness devices are defined as fitness bracelets or fitness watches.

Patients are one of the main target groups for ICTs related to health care. The use of ICTs can increase patients’ enlightened participation by enabling them to take more active control of their own health [4]. ICTs can enable better self-assessment of health status and increase patient safety and involvement [5].

However, competencies and skills are necessary for patients to use ICTs to improve their own health. In this context, health literacy (HL) is important [6]. In the context of the COVID-19 pandemic and the associated spread of health-related misinformation and widespread implementation of digital health services such as video consults, the concept of HL has become increasingly important [7,8]. Particularly in the context of the increasing significance of ICTs in general and mobile apps in particular, eHealth literacy (eHL) has become an even more important concept than HL [9]. Incorporated into this concept are the ability to navigate information on the internet and the ability to self-manage ICT use. In addition, eHL includes analytical competencies [10-12]. Another concept that is closely related to eHL is digital health equity. This describes the differences in the access to and use of ICTs between different populations and groups. This concept is of greatest interest regarding the increasing provision of digital health care as it describes the possible exclusion of people who lack access to and the ability to use ICTs [2,13].

The increased use of ICTs in various areas of life that occurred during the pandemic in the context of social distancing [14] has had significant implications for the issues considered in this paper.

### Objectives

During the COVID-19 pandemic, there has been a tremendous increase in the use of eHealth technologies, such as video consultations [15]. However, this trial was conducted in the pre–COVID-19 era and provides an overview of the perception of these new technologies in a representative cohort of surgical patients. The aim of this pilot study was to illustrate the patient-centered aspects of digital health technologies and their use, dissemination, and acceptance depending on basic patient-related sociodemographic data. Acceptance is deﬁned as the intention to use and, in some cases, as the actual use of health technologies [16,17]. Therefore, we investigated how surgical patients access and use digital health technologies and how their use behavior differs among different population subgroups.

## Methods

### Study Design

This single-center, cross-sectional survey study (Medical Information and Communication Technology Use of Patients) was conducted at the University Hospital Dresden, Germany, in the Department of Visceral, Thoracic, and Vascular Surgery between September 2019 and March 2020. A self-administered questionnaire was developed in consultation with a statistician and an expert in data management (Multimedia Appendix 1). The questionnaire was pretested and validated in a group of 10 persons working at the surgical trial unit in the Department of Surgery, University Hospital Dresden. The questionnaire was provided as a paper-based survey to ensure the participation of all patients. As the main aim of this pilot trial was to obtain a status quo of the use and perception of eHealth and mHealth technologies in a representative cohort of surgical patients rather than a thorough investigation of a general technology acceptance model, we chose to design a short, nonvalidated questionnaire ourselves. Questions were partly taken from existing instruments and partly added after obtaining results from structured qualitative interviews with patients and surgeons before this trial [9,18-20].

### Ethics Approval

This study was conducted in line with the principles of the Declaration of Helsinki. The trial was approved by the ethics committee (Institutional Review Board) of the Technical University Dresden (file 313062019, June 7, 2019). The American Association for Public Opinion Research guidelines were followed as applicable.

### Measurements

The questionnaire contained four categories: (1) questions on general information, (2) questions on the use of the internet, (3) questions on mobile phones (cell phones) and smartphones, and (4) general questions on the use of ICTs (Multimedia Appendix 1). Participant-related and demographic data were collected in the questionnaire. The survey was anonymous, and a pooled analysis of the questionnaire responses was conducted so that no conclusions could be drawn about individual participants.

### Sampling and Recruitment

Questionnaires were distributed to randomly selected nonemergency patients at the receptionist’s office during the first patient contact at the Department of Surgery at the Dresden University Hospital, Germany.

The inclusion criteria to participate in the study were (1) admission to the clinic of the Department of Visceral, Thoracic, and Vascular Surgery of the University Hospital Dresden; (2) being aged ≥18 years; (3) provision of verbal consent; and (4) understanding of the German language owing to the presentation of the questionnaire in German.

The exclusion criteria were (1) admission to the hospital because of an emergency, (2) language problems, and (3) an impaired mental state or lack of compliance.

### Data Collection

After oral information was provided and individuals were asked to participate in the study and complete the questionnaire, the participants were presented with the questionnaire. The participants were informed that their refusal to participate would not result in any disadvantages. The time needed to complete the questionnaire was approximately 15 minutes. No compensation was offered.

### Data Analysis

Data from the questionnaires were pooled into a table using Microsoft Excel (Microsoft Office 2016). Demographic data analyses and statistical analyses were performed using R (version 4.0.2; R Foundation for Statistical Computing) in RStudio (version 1.2.1335; RStudio, Inc). A chi-square test was performed to show any relationship between the age group and the variables under consideration (*P*<.05).

## Results

### Overview

In total, 650 questionnaires were distributed to randomly selected nonemergency patients at the receptionist’s office during the first patient contact. Of these, 406 questionnaires were returned, yielding a response rate of 62.5% (406/650). However, not all participants answered all questions, as can be seen in the results tables, which provide the absolute numbers for each question.

### Participant Characteristics

Table 1 shows the participants’ demographic data and general information. The participants were divided into 3 age groups (young adults: 18-40 years; middle-aged adults: 41-70 years; and older adults: >71 years) to investigate differences according to age. In total, 56.1% (226/403) were men and 43.9% (177/403) were women; 15.1% (61/405) were young adults, 63% (255/405) were middle-aged adults, and 22% (89/405) were older adults. Only 26.8% (102/381) of the participants lived in cities with >100,000 inhabitants. Of the 406 participants, 353 (86.9%) visited their general practitioner on a regular basis, whereas only 22 (5.4%) did not regularly visit any physician. A total of 14% (55/394) reported >10 outpatient and hospital visits per year. In the sample population, 32% (123/384) had obtained an academic degree as the highest form of education, 56.8% (218/384) had completed vocational training, and 4.7% (18/384) had a school-leaving degree (see Table S1 in Multimedia Appendix 2 for the patients’ demographic data by level of education). In total, 58.9% (229/389) of the participants had a chronic disease.

**Table 1:** Participants’ demographic data sorted by age group (N=406).

Characteristic		Age 18 to 40 years (n=61), n (%)	Age 41 to 70 years (n=255), n (%)	Age ≥71 years (n=89), n (%)	Total^a^, n (%)
**Sex**					
	Male	31 (50.8)	145 (57.1)	50 (56.8)	226 (56.1)
	Female	30 (49.2)	109 (42.9)	38 (43.2)	177 (43.9)
	Total	61 (100)	254 (100)	88 (100)	403 (100)
**Inhabitants in hometown**					
	<10,000	11 (18.6)	96 (39.7)	25 (31.2)	132 (34.6)
	10,000 to 50,000	13 (22)	58 (24)	24 (30)	95 (24.9)
	50,000 to 100,000	2 (3.4)	11 (4.5)	6 (7.5)	19 (5)
	>100,000	29 (49.2)	58 (24)	15 (18.8)	102 (26.8)
	Not known	4 (6.8)	19 (7.9)	10 (12.5)	33 (8.7)
	Total	59 (100)	242 (100)	80 (100)	381 (100)
**Regularly visited physicians^b^**					
	None	13 (21.3)	9 (3.5)	0 (0)	22 (5.4)
	General practitioner	41 (67.2)	227 (89)	84 (94.4)	353 (86.9)
	Cardiologist	4 (6.6)	23 (9)	20 (22.5)	48 (11.8)
	Gastroenterologist	7 (11.5)	20 (7.8)	10 (11.2)	37 (9.1)
	Other physician	18 (29.5)	86 (33.7)	34 (38.2)	138 (34)
	Total	61 (100)	255 (100)	89 (100)	406 (100)
**Outpatient and hospital visits per year**					
	Never	1 (1.6)	5 (2)	0 (0)	6 (1.5)
	1	6 (9.8)	22 (8.9)	3 (3.4)	31 (7.9)
	2 to 3	31 (50.8)	82 (33.3)	18 (20.7)	131 (33.2)
	4 to 6	10 (16.4)	75 (30.5)	39 (44.8)	124 (31.5)
	7 to 9	5 (8.2)	28 (11.4)	14 (16.1)	47 (11.9)
	≥10	8 (13.1)	34 (13.8)	13 (14.9)	55 (14)
	Total	61 (100)	246 (100)	87 (100)	394 (100)
**Degree obtained**					
	Still in training	8 (13.1)	0 (0)	1 (1.3)	9 (2.3)
	School-leaving certificate	2 (3.3)	10 (4.1)	6 (7.7)	18 (4.7)
	Completed vocational training	34 (55.7)	150 (61.5)	33 (42.3)	218 (56.8)
	Completed academic degree	16 (26.2)	76 (31.1)	31 (39.7)	123 (32)
	No degree or no training	0 (0)	2 (0.8)	0 (0)	2 (0.5)
	Not specified	1 (1.6)	6 (2.5)	7 (9)	14 (3.6)
	Total	61 (100)	244 (100)	78 (100)	384 (100)
**Chronic disease**					
	Yes	25 (41.7)	144 (59.8)	60 (69)	229 (58.9)
	No	35 (58.3)	97 (40.2)	27 (31)	160 (41.1)
	Total	60 (100)	241 (100)	87 (100)	389 (100)

^a^Includes questionnaire responses without information on the age group provided, which are therefore only counted in the *Total* column.

^b^Multiple answers were possible, and the percentage refers to the respective total.

### Participant Internet Use by Age Group

Table 2 shows the internet use of the participants by age group. In total, 67% (266/397) of the participants reported having searched the internet for information about diseases at any time in the past. There was a statistically significant difference (*P*=.004) by age group, with 34% (29/85) of the older adults, 71.8% (181/252) of the middle-aged adults, and 93% (56/60) of the young adults using the internet to search for information about diseases (266/397, 67%). Almost half of the participants (180/390, 46.2%) had searched the internet for information about their current illness before their hospital visit. This was done by more young adults than middle-aged adults and older adults, resulting in a strong relationship with the respective age groups (*P*<.001). Most participants (249/406, 61.3%) indicated that they had taught themselves how to use computers and smartphones, whereas some of the middle-aged adults and older adults (5/255, 2% and 4/89, 5%) had visited adult education centers for this purpose. A total of 24.6% (96/390) of the participants did not have access to broadband internet at home. The proportion of older adults who did not have access to broadband internet (37/81, 46%) was significantly higher than that of young and middle-aged adults (*P*<.001).

**Table 2:** Participant internet use sorted by age group (N=406).

Characteristic			Age 18 to 40 years (n=61), n (%)		Age 41 to 70 years (n=255), n (%)		Age ≥71 years (n=89), n (%)		Total^a^, n (%)
**Searches the internet for diseases in general**									
	Yes	56 (93.3)		181 (71.8)		29 (34.1)		266 (67)	
	No	4 (6.7)		69 (27.4)		55 (64.7)		128 (32.2)	
	Not known	0 (0)		2 (0.8)		1 (1.2)		3 (0.8)	
	Total	60 (100)		252 (100)		85 (100)		397 (100)	
**Searches for web-based information about current illness**									
	Yes	38 (63.3)		116 (47)		26 (31.3)		180 (46.2)	
	No	22 (36.7)		131 (53)		57 (68.7)		210 (53.8)	
	Total	60 (100)		247 (100)		83 (100)		390 (100)	
**Learning to use computers or smartphones^b^**									
	Self-taught	57 (93.4)		169 (66.3)		23 (25.8)		249 (61.3)	
	Internet research	8 (13.1)		17 (6.7)		0 (0)		25 (6.2)	
	Family or friends	17 (27.9)		108 (42.4)		23 (25.8)		148 (36.5)	
	Adult education center	0 (0)		5 (2)		4 (4.5)		9 (2.2)	
	Other	3 (4.9)		18 (7.1)		9 (10.1)		30 (7.4)	
	Total	61 (100)		255 (100)		89 (100)		406 (100)	
**DSL^c^ or broadband connection at home**									
	Yes	52 (88.1)		178 (71.2)		38 (46.9)		268 (68.7)	
	No	4 (6.8)		55 (22)		37 (45.7)		96 (24.6)	
	Not known	3 (5.1)		17 (6.8)		6 (7.4)		26 (6.7)	
	Total	59 (100)		250 (100)		81 (100)		390 (100)	

^a^Includes questionnaire responses without information on the age group provided, which are therefore only counted in the *Total* column.

^b^Multiple answers were possible, and the percentage refers to the respective total.

^c^DSL: digital subscriber line.

### Use of Mobile Phones and Smartphones

Table 3 summarizes the use of mobile phones and smartphones by age group. In total, 7.9% (31/393) of the study population did not own a cell phone. The percentage of participants who did not own a mobile phone was significantly higher in the group of older adults (20/89, 24%; *P*<.001), whereas all young adults owned a mobile phone. A total of 78.9% (296/375) of the respondents reported that their mobile phones were smartphones, and the proportion of older adults who did not own a smartphone was significantly higher (35/72, 49%; *P*<.001). The Android operating system was used by most participants (192/330, 58.2%) followed by the Apple iOS system (77/330, 23.3%). Of the 49 older adults, 19 (39%) did not know which operating system was installed on their mobile phones. Most participants (299/364, 82.1%) did not own a fitness device. Significantly more young adults than older adults owned such a device (*P*<.001). Table 3 shows the participants’ typical use of their smartphones in the different age groups: 12.2% (45/369) of the participants already used medical and health apps, whereas 20% (12/60) of the young adults and 4% (3/75) of the older adults used medical and health apps (*P*=.005).

**Table 3:** Participants’ use of mobile phones, cell phones, or smartphones sorted by age group (N=406).

Characteristic		Age 18 to 40 years (n=61), n (%)	Age 41 to 70 years (n=255), n (%)	Age ≥71 years (n=89), n (%)	Total^a^, n (%)
**Owns a mobile phone**					
	Yes	60 (100)	238 (95.6)	64 (76.2)	362 (92.1)
	No	0 (0)	11 (4.4)	20 (23.8)	31 (7.9)
	Total	60 (100)	249 (100)	84 (100)	393 (100)
**Mobile phone is a smartphone**					
	Yes	60 (100)	204 (84)	32 (44.4)	296 (78.9)
	No	0 (0)	36 (14.8)	35 (48.6)	71 (18.9)
	Not known	0 (0)	3 (1.2)	5 (6.9)	8 (2.1)
	Total	60 (100)	243 (100)	72 (100)	375 (100)
**Operating system of the smartphone**					
	iOS	20 (33.3)	54 (24.4)	3 (6.1)	77 (23.3)
	Android	38 (63.3)	133 (60.2)	21 (42.9)	192 (58.2)
	Miscellaneous	1 (1.7)	13 (5.9)	6 (12.2)	20 (6.1)
	Not known	1 (1.7)	21 (9.5)	19 (38.8)	41 (12.4)
	Total	60 (100)	221 (100)	49 (100)	330 (100)
**Owns a fitness device**					
	No	40 (66.7)	198 (84.6)	61 (87.1)	299 (82.1)
	Fitness bracelet or smartwatch	19 (31.7)	29 (12.4)	5 (7.1)	53 (14.6)
	Yes, other	1 (1.7)	7 (3)	4 (5.7)	12 (3.3)
	Total	60 (100)	234 (100)	70 (100)	364 (100)
**Uses a smartphone or mobile phone for...^b^**					
	Phone calls	52 (85.2)	223 (87.5)	54 (60.7)	329 (81)
	Messenger services or SMS	58 (95.1)	171 (67.1)	24 (27)	253 (62.3)
	Social media	45 (73.8)	62 (24.3)	3 (3.4)	110 (27.1)
	Route planning and navigation	51 (83.6)	125 (49)	13 (14.6)	189 (46.6)
	Medical or health apps	18 (29.5)	33 (12.9)	6 (6.7)	57 (14)
	Photography and photo use	54 (88.5)	162 (63.5)	27 (30.3)	243 (59.9)
	Listening to music	43 (70.5)	61 (23.9)	1 (1.1)	105 (25.9)
	Watching movies and series	29 (47.5)	20 (7.8)	2 (2.2)	51 (12.6)
	Web browsing	53 (86.9)	121 (47.5)	11 (12.4)	185 (45.6)
	Games	25 (41)	39 (15.3)	6 (6.7)	70 (17.2)
	None or not applicable	0 (0)	0 (0)	4 (4.5)	4 (1)
	Other	1 (1.6)	4 (1.6)	1 (1.1)	6 (1.5)
	Total	61 (100)	255 (100)	89 (100)	406 (100)
**Use of apps related to health**					
	Yes	12 (20)	30 (12.8)	3 (4)	45 (12.2)
	No	45 (75)	202 (86.3)	68 (90.7)	315 (85.4)
	Not known or not applicable	3 (5)	2 (0.9)	4 (5.3)	9 (2.4)
	Total	60 (100)	234 (100)	75 (100)	369 (100)

^a^Includes questionnaire responses without information on the age group provided, which are therefore only counted in the *Total* column.

^b^Multiple answers were possible, and the percentage refers to the respective total.

### Use of ICTs by the Participants

Table 4 summarizes the participants’ use of and attitudes toward ICTs used in medicine, sorted by age group. Merely 30% (112/373) of the participants thought it was useful to introduce web-based or video consultations. Almost as many participants (100/373, 26.8%) indicated that they did not know. Again, there were significant differences by age group, as shown in Table 4 (*P*<.001): 74.2% (276/372) of the participants considered electronic health records to be useful, with a statistically significant difference by age group (*P*=.001). Only 1.7% (6/350) of the participants would mostly trust an app to make a correct decision, 48.3% (169/350) would trust a physician, 22.6% (79/350) would trust none of the options, and 27.4% (96/350) did not know. The main disadvantages identified for video consultations were the lack of personal contact (225/406, 55.4%) and the absence of physical examination (264/406, 65%). Many participants stated that they would never take advantage of a video consultation (130/342, 38%). Only 5% (17/342) stated that they would use a video consultation as often as possible. A statistically significant difference by age group (*P*=.01), with older adults least wanting to take advantage of video consultations, was also observed for this aspect. Many participants (176/355, 49.6%) did not believe that the use of a fitness device could improve or enhance their health, whereas 26.8% (95/355) did not know. A significant difference among the age groups was found, with *P*<.001.

**Table 4:** Participants’ use of information and communication technologies sorted by age group (N=406).

Question		Age 18 to 40 years (n=61), n (%)	Age 41 to 70 years (n=255), n (%)	Age ≥71 years (n=89), n (%)	Total^a^, n (%)
**Do you think it would be useful to introduce web-based consultations?**					
	Yes	28 (46.7)	74 (31.6)	10 (12.7)	112 (30)
	No	20 (33.3)	99 (42.3)	42 (53.2)	161 (43.2)
	Not known	12 (20)	61 (26.1)	27 (34.2)	100 (26.8)
	Total	60 (100)	234 (100)	79 (100)	373 (100)
**Do you consider an electronic health record to be basically useful?**					
	Yes	51 (85)	181 (77)	44 (57.1)	276 (74.2)
	No	5 (8.3)	20 (8.5)	16 (20.8)	41 (11)
	Not known	4 (6.7)	34 (14.5)	17 (22.1)	55 (14.8)
	Total	60 (100)	235 (100)	77 (100)	372 (100)
**Do you trust...to make a correct diagnosis?**					
	An app	4 (6.8)	0 (0)	2 (3)	6 (1.7)
	A physician (on the web)	27 (45.8)	120 (53.3)	22 (33.3)	169 (48.3)
	None	10 (16.9)	48 (21.3)	21 (31.8)	79 (22.6)
	Not known	18 (30.5)	57 (25.3)	21 (31.8)	96 (27.4)
	Total	59 (100)	225 (100)	66 (100)	350 (100)
**Do you see disadvantages of a video consultation with a telemedicine provider?^b^**					
	No disadvantages	4 (6.6)	12 (4.7)	4 (4.5)	20 (4.9)
	Lack of personal contact	33 (54.1)	147 (57.6)	45 (50.6)	225 (55.4)
	No physical examination	47 (77)	176 (69)	41 (46.1)	264 (65)
	Physician unknown or anonymous	22 (36.1)	90 (35.3)	19 (21.3)	131 (32.3)
	Lack of confidence in the competence of the physician	20 (32.8)	74 (29)	11 (12.4)	105 (25.9)
	No prescription of medication possible	26 (42.6)	89 (34.9)	24 (27)	139 (34.2)
	Unsecure internet connection	31 (50.8)	105 (41.2)	14 (15.7)	150 (36.9)
	Other	1 (1.6)	2 (0.8)	1 (1.1)	4 (1)
	Total	61 (100)	255 (100)	89 (100)	406 (100)
**Would you take advantage of a video consultation in medical care?**					
	As often as possible	7 (11.9)	9 (4.1)	1 (1.6)	17 (5)
	Frequently	8 (13.6)	28 (12.6)	3 (4.9)	39 (11.4)
	Rather rarely	17 (28.8)	67 (30.2)	12 (19.7)	96 (28.1)
	Not at all	20 (33.9)	83 (37.4)	27 (44.3)	130 (38)
	Not known	7 (11.9)	35 (15.8)	18 (29.5)	60 (17.5)
	Total	59 (100)	222 (100)	61 (100)	342 (100)
**Would the use of a fitness bracelet or a smartwatch improve or enhance your health?**					
	Yes, very much	9 (15.5)	7 (3.1)	1 (1.5)	17 (4.8)
	Yes, a little bit	13 (22.4)	46 (20.1)	8 (11.8)	67 (18.9)
	No	24 (41.4)	117 (51.1)	35 (51.5)	176 (49.6)
	Not known	12 (20.7)	59 (25.8)	24 (35.3)	95 (26.8)
	Total	58 (100)	229 (100)	68 (100)	355 (100)

^a^Includes questionnaire responses without information on the age group provided, which are therefore only counted in the *Total* column.

^b^Multiple answers were possible, and the percentage refers to the respective total.

### Use and Perceived Usefulness of Medical and Health Apps

Table 5 shows the assessment of use behavior and the perceived usefulness of medical and health apps. Only between 46.1% (187/406) and 49% (199/406) of the participants answered the questions in this section. Approximately half of all respondents rated the usefulness of various apps positively, including apps for medication (110/195, 56.4%), monitoring of vital signs (97/199, 48.7%), web-based appointments (111/189, 58.7%), exchanges with health insurance companies (91/187, 48.7%), and fitness (77/196, 39.3%). However, 19.4% (38/196) of the participants were already using such an app. Again, there were differences among the age groups. Very few participants in the older adult age group were able to answer the questions in this section.

**Table 5:** Use and usefulness of medical apps for participants by age group (N=406).

Characteristic		Age 18 to 40 years (n=61), n (%)	Age 41 to 70 years (n=255), n (%)	Age ≥71 years (n=89), n (%)	Total, n (%)
**Medication app (eg, reminders or insulin scheme)**					
	Finding it useful	34 (82.9)	67 (52.8)	9 (33.3)	110 (56.4)
	Using it	2 (4.9)	17 (13.4)	4 (14.8)	23 (11.8)
	Not useful	5 (12.2)	43 (33.9)	14 (51.9)	62 (31.8)
	Total	41 (100)	127 (100)	27 (100)	195 (100)
**App for monitoring of vital signs (eg, pulse or blood sugar)**					
	Finding it useful	32 (72.7)	57 (43.5)	8 (33.3)	97 (48.7)
	Using it	3 (6.8)	20 (15.3)	5 (20.8)	28 (14.1)
	Not useful	9 (20.5)	54 (41.2)	11 (45.8)	74 (37.2)
	Total	44 (100)	131 (100)	24 (100)	199 (100)
**Web-based appointment allocation and coordination app**					
	Finding it useful	30 (71.4)	71 (56.8)	10 (45.5)	111 (58.7)
	Using it	6 (14.3)	21 (16.8)	3 (13.6)	30 (15.9)
	Not useful	6 (14.3)	33 (26.4)	9 (40.9)	48 (25.4)
	Total	42 (100)	125 (100)	22 (100)	189 (100)
**App of the health insurance company with access to information such as patient data, findings, and vaccination status**					
	Finding it useful	31 (70.5)	50 (42)	10 (41.7)	91 (48.7)
	Using it	4 (9.1)	16 (13.4)	4 (16.7)	24 (12.8)
	Not useful	9 (20.5)	53 (44.5)	10 (41.7)	72 (38.5)
	Total	44 (100)	119 (100)	24 (100)	187 (100)
**Fitness app for recording physical activity**					
	Finding it useful	26 (57.8)	48 (36.9)	3 (14.3)	77 (39.3)
	Using it	9 (20)	23 (17.7)	6 (28.6)	38 (19.4)
	Not useful	10 (22.2)	59 (45.4)	12 (57.1)	81 (41.3)
	Total	45 (100)	130 (100)	21 (100)	196 (100)

### Analysis by Education Status Obtained

We analyzed differences in results according to academic degree (Tables S2-S5 in Multimedia Appendix 2). There was a significant difference by academic degree in the percentage of patients searching on the web for information about their current illness (Table S2 in Multimedia Appendix 2). Although 60.3% (73/121) of the patients with an academic degree had searched for information regarding their illness before the hospital visit, only 41.7% (100/240) of the patients with another degree or no degree had done so (*P*=.001). There were also significant differences in the perception of electronic health records as useful (*P*=.01) and in trust in an app, a physician, or none of the options to make a correct diagnosis (*P*=.01). A total of 65% (47/72) of the participants with an academic degree perceived a web-based appointment allocation tool to be useful (compared with 63/110, 57.3% of the participants with no degree), and 21% (15/72) were already using such an app (compared with only 14/110, 12.7% of the participants with another degree or no degree; *P*=.03; Table S5 in Multimedia Appendix 2).

## Discussion

### Principal Findings

In a cohort of 406 patients from the outpatient department of a tertiary academic referral center in Germany, we found significant differences in the use and perception of digital health technologies depending on the age and educational status of the patients. Older patients and patients with a lower education status lack access to broadband internet and knowledge of and access to smartphone use; these populations have a lower level of eHL.

The aim of this trial was not an in-depth analysis of use and acceptance models of modern ICTs but rather to obtain a status quo of the actual use and perception of modern eHealth and mHealth technologies in a representative cohort of surgical patients in Germany in the pre–COVID-19 era.

Overall, the older the participants were, the more frequently they had regular contact with physicians and were hospitalized. Unfortunately, fewer people of older age answered the questions. We suspect that many of these people did not understand the specific topic or could not answer the questions. However, the older the patients were, the less frequently they searched for information on the internet about illness in general or their own illnesses specifically and the less ready they were to use ICTs with regard to their health [21-24]. This finding is in line with previous data reporting that information technology (IT) use and IT use for health are more common and better accepted by younger people [25]. This indicates a clear difficulty faced by older people with regard to their involvement in health decisions if up-to-date health information on the internet is accessible [26]. Six access points for seeking health information have been classified: (1) interpersonal sources, (2) traditional mass media, (3) traditional and modern internet sources, (4) Web 2.0 sources, (5) libraries, and (6) government agencies or social services [27]. Which of these access points people use depends not only on the design and accessibility of the source itself but also on the users [27].

The older the participants in our study were, the less likely they were to have learned how to use smartphones and computers themselves and the more they relied on external help. This is closely related to the digital literacy paradox described in the literature: competencies for using ICTs can be learned only by using ICTs. However, older people often do not have full access to ICTs and continue to have poor digital literacy skills [28]. This is consistent with the results of our study, which revealed that only a minority of the older adults reported owning smartphones and <50% of them (38/81, 47%) had access to broadband connections at home. However, it was not only older people who showed a limited understanding of ICTs in our study. For example, 12.4% (41/330) of the participants said they did not know which operating system their smartphones had. In 2020, the European Union Memorandum on Lifelong Learning explicitly included IT skills among the new basic skills that all individuals need [29]. This underscores the danger of people being excluded from a large area of (social) life owing to a lower level of competence in this area [30].

A recent trial found that low HL was related to limited functional HL, low socioeconomic status, and frequent visits to physicians [31]. These findings indicate a strong need for HL education services across all age groups.

Other findings of this study were that the participants were partially unaware of the possibility of ICTs and the participants’ confidence in the usefulness of medical and health apps was low. It is also noticeable that not all participants answered these questions. This indicates that this technology is rarely used. Presumably, this also has an impact on trust in it. However, many of the participants also stated that they were unable to make a statement in this regard, and only 48.3% (169/350) of all respondents believed that a physician could make a correct diagnosis on the web. Certainly, occasions exist in which a face-to-face consultation is essential. However, many of the drawbacks cited by the participants were unfounded [22-24,32]. These findings have been further reinforced in the context of the increase in telemedicine during the COVID-19 pandemic [33].

Considering that 58.9% (229/389) of the participants had a chronic illness, the low number of people using medical and health apps is surprising. Current data show positive results when medical and health apps are used by patients with chronic diseases. However, for some apps, no evidence of a benefit exists [34]. Regardless of whether they are used for chronic disease management, medical and health apps can confer benefits [35]. The literature shows that the use of medical and health apps can have positive consequences but that their use must always be critically questioned. There are 4 factors playing a significant role as direct determinants of user acceptance and use behavior: performance expectancy, effort expectancy, social influence, and facilitating conditions.

Relatedly, it is important to consider that the use of ICTs also poses dangers that users should be aware of to protect themselves. Younger participants used new technologies in private and health care settings more often and evaluated the benefits more positively than older participants. In general, younger people are more positive about health information from the internet than older people [36]. Nevertheless, many young adults in our study reported concerns about data security when using ICTs. These concerns are entirely justified. The ICT market for health care is currently opaque. ICTs often use sensitive data for other purposes [37]. Therefore, it is more worrying that the participants in the other age groups indicated significantly lower concerns in this regard. The study also showed that, in general, people with a higher education status were more informed, were more open, and used modern ICTs more frequently than people with a lower education status. This finding highlights the fact that the digital divide is related not only to the age group to which patients belong but also to other socioeconomic aspects such as educational status [38].

However, educating patients regarding eHL alone is not sufficient to improve the quality of care. Health care professionals must also be trained to provide meaningful information and guidance to patients. Currently, eHL among physicians is limited [39]. Therefore, it is necessary to pursue an approach that enables education for the entirety of society [40]. It is of utmost importance to focus on the digital divide among different age and socioeconomic groups in the design and implementation of new digital technologies [41].

### Limitations

In some ways, this paper is anachronistic, reflecting results regularly reported 10 to 20 years ago for other countries or for industries other than health care. The reason for this is that remote medical consultations were not permitted in Germany until 2018. Another limitation of this study from the current perspective is the timing of the survey before the COVID-19 pandemic. The pandemic has led to considerable changes in health care, especially regarding the use of telemedicine [42] as well as the use of apps for contact tracing [43] and self-reported symptom tracking [44]. The COVID-19 pandemic has changed people’s views; in a recent study of patients of urology in Germany, 85% wanted a videoconference teleconsultation rather than a face-to-face consultation [45]. However, the potential impact on eHL requires further investigation. The pandemic may also have had an impact on the digital divide as many individuals have experienced pay cuts or job losses because of the pandemic [46].

However, the pandemic has also highlighted the critical importance of the study described in this paper. For the routine care of patients, ICTs have been widely used and implemented during the COVID-19 crisis [47]. Good HL is the foundation for understanding the risk factors and consequences of contagion during the COVID-19 pandemic [48]. Thus, the pandemic can hopefully become the driving force for much-needed improvements in eHL that can support digital equity.

In our opinion, nonresponse bias does not threaten the validity of the findings of this study. It is likely that the results would be even more pronounced as people with limited eHL mostly refused to participate and did not answer the questions. This was randomly confirmed by routinely questioning patients who decided not to participate about their reasons.

We did not use validated instruments or methods for user technology acceptance in this work, such as the unified theory of acceptance and use of technology model, as our focus was not on a thorough technology acceptance investigation but rather on obtaining a general status quo of use, attitudes, and behavior of surgical patients in terms of new digital health technologies. However, recently, much work has been done regarding digitization and digital health technology in Germany, which we will use to design future trials [49-51]. For further studies, it would be recommended to use additional validated instruments.

### Conclusions

The data from our study indicate that there is a need for education across all age groups regarding the opportunities and risks of using ICTs in health care. Regarding eHL, it is important that educational programs build participants’ knowledge in the areas of computers and smartphones, health, and science. The increase in the importance of ICTs can promote participative decisions in health care, enabling patients to influence their illnesses and drive prevention via active self-management. However, critical to the provision of such health care is the training and equipping of all patients, especially older patients, to enable them to safely use ICTs.

With respect to medical and health apps focused on the self-management of chronic conditions, app developers should be mandated to use data-based evidence to increase the safety and usefulness of the apps. Further research should address patient needs that must be met for patients to be able to actively use health-related ICTs. In addition, it is crucial to investigate how trust in the use of ICTs in the health care setting can be increased [52,53].

## Appendix

Multimedia Appendix 1 Original questionnaire.

Multimedia Appendix 2 Participants’ demographic data and analyses by degree obtained.

## Abbreviations

eHL: eHealth literacy
HL: health literacy
ICT: information and communication technology
IT: information technology
mHealth: mobile health

## References

[1] Kernebeck S, Busse TS, Böttcher MD, Weitz J, Ehlers J, Bork U (2020). Impact of mobile health and medical applications on clinical practice in gastroenterology. World J Gastroenterol.

[2] Rodriguez JA, Clark CR, Bates DW (2020). Digital health equity as a necessity in the 21st century cures act era. JAMA.

[3] da Fonseca MH, Kovaleski F, Picinin CT, Pedroso B, Rubbo P (2021). E-health practices and technologies: a systematic review from 2014 to 2019. Healthcare (Basel).

[4] Lux T, Breil B, Dörries M, Gensorowsky D, Greiner W, Pfeiffer D, Rebitschek FG, Gigerenzer G, Wagner GG (2017). [Digitalisierung im Gesundheitswesen — zwischen Datenschutz und moderner Medizinversorgung]. Wirtschaftsdienst.

[5] Wangler J, Jansky M (2020). [Gesundheits-Apps als Instrumente der Prävention? – Eine Interviewstudie zu Potenzialen für das hausärztliche Setting]. Präv Gesundheitsf.

[6] Sørensen K, Van den Broucke S, Fullam J, Doyle G, Pelikan J, Slonska Z, Brand H, (HLS-EU) Consortium Health Literacy Project European (2012). Health literacy and public health: a systematic review and integration of definitions and models. BMC Public Health.

[7] Chong YY, Cheng HY, Chan HY, Chien WT, Wong SY (2020). COVID-19 pandemic, infodemic and the role of eHealth literacy. Int J Nurs Stud.

[8] Liu M, Caputi TL, Dredze M, Kesselheim AS, Ayers JW (2020). Internet searches for unproven COVID-19 therapies in the United States. JAMA Intern Med.

[9] Norman CD, Skinner HA (2006). eHEALS: the eHealth literacy scale. J Med Internet Res.

[10] Neter E, Brainin E (2019). Association between health literacy, eHealth literacy, and health outcomes among patients with long-term conditions. Eur Psychol.

[11] Guo SH, Hsing H, Lin J, Lee C (2021). Relationships between mobile eHealth literacy, diabetes self-care, and glycemic outcomes in taiwanese patients with type 2 diabetes: cross-sectional study. JMIR Mhealth Uhealth.

[12] Stellefson M, Paige SR, Alber JM, Chaney BH, Chaney D, Apperson A, Mohan A (2019). Association between health literacy, electronic health literacy, disease-specific knowledge, and health-related quality of life among adults with chronic obstructive pulmonary disease: cross-sectional study. J Med Internet Res.

[13] Sun GH (2012). The digital divide in internet-based patient education materials. Otolaryngol Head Neck Surg.

[14] De' R, Pandey N, Pal A (2020). Impact of digital surge during Covid-19 pandemic: a viewpoint on research and practice. Int J Inf Manage.

[15] Doraiswamy S, Abraham A, Mamtani R, Cheema S (2020). Use of telehealth during the COVID-19 pandemic: scoping review. J Med Internet Res.

[16] Venkatesh V, Morris MG, Davis GB, Davis FD (2003). User acceptance of information technology: toward a unified view. MIS Quarterly.

[17] Davis FD (1989). Perceived usefulness, perceived ease of use, and user acceptance of information technology. MIS Quarterly.

[18] Marsall M, Engelmann G, Skoda E, Teufel M, Bäuerle A (2022). Measuring electronic health literacy: development, validation, and test of measurement invariance of a revised German version of the eHealth literacy scale. J Med Internet Res.

[19] Lee J, Lee E, Chae D (2021). eHealth literacy instruments: systematic review of measurement properties. J Med Internet Res.

[20] Salgado T, Tavares J, Oliveira T (2020). Drivers of mobile health acceptance and use from the patient perspective: survey study and quantitative model development. JMIR Mhealth Uhealth.

[21] Park S, Kim B (2020). Predictors of internet use among older adults with diabetes in South Korea: survey study. JMIR Med Inform.

[22] Eberly LA, Kallan MJ, Julien HM, Haynes N, Khatana SA, Nathan AS, Snider C, Chokshi NP, Eneanya ND, Takvorian SU, Anastos-Wallen R, Chaiyachati K, Ambrose M, O'Quinn R, Seigerman M, Goldberg LR, Leri D, Choi K, Gitelman Y, Kolansky DM, Cappola TP, Ferrari VA, Hanson CW, Deleener ME, Adusumalli S (2020). Patient characteristics associated with telemedicine access for primary and specialty ambulatory care during the COVID-19 pandemic. JAMA Netw Open.

[23] Lam K, Lu AD, Shi Y, Covinsky KE (2020). Assessing telemedicine unreadiness among older adults in the United States during the COVID-19 pandemic. JAMA Intern Med.

[24] Wright JP, Edwards GC, Goggins K, Tiwari V, Maiga A, Moses K, Kripalani S, Idrees K (2018). Association of health literacy with postoperative outcomes in patients undergoing major abdominal surgery. JAMA Surg.

[25] Paslakis G, Fischer-Jacobs J, Pape L, Schiffer M, Gertges R, Tegtbur U, Zimmermann T, Nöhre M, de Zwaan M (2019). Assessment of use and preferences regarding internet-based health care delivery: cross-sectional questionnaire study. J Med Internet Res.

[26] Lee HY, Jin SW, Henning-Smith C, Lee J, Lee J (2021). Role of health literacy in health-related information-seeking behavior online: cross-sectional study. J Med Internet Res.

[27] Zhang Y (2014). Beyond quality and accessibility: source selection in consumer health information searching. J Assn Inf Sci Tec.

[28] Schreurs K, Quan-Haase A, Martin K (2017). Problematizing the digital literacy paradox in the context of older adults’ ICT use: aging, media discourse, and self-determination. Can J Commun.

[29] A memorandum on lifelong learning. VOCED Plus.

[30] Schmidt-Hertha B, Strobel-Dümer C (2014). Computer literacy among the generations: how can older adults participate in digital society?. Challenging the 'European Area of Lifelong Learning'.

[31] Berens E, Vogt D, Messer M, Hurrelmann K, Schaeffer D (2016). Health literacy among different age groups in Germany: results of a cross-sectional survey. BMC Public Health.

[32] Asiri A, AlBishi S, AlMadani W, ElMetwally A, Househ M (2018). The use of telemedicine in surgical care: a systematic review. Acta Inform Med.

[33] Mann DM, Chen J, Chunara R, Testa PA, Nov O (2020). COVID-19 transforms health care through telemedicine: evidence from the field. J Am Med Inform Assoc.

[34] Scott IA, Scuffham P, Gupta D, Harch TM, Borchi J, Richards B (2020). Going digital: a narrative overview of the effects, quality and utility of mobile apps in chronic disease self-management. Aust Health Rev.

[35] Carroll JK, Moorhead A, Bond R, LeBlanc WG, Petrella RJ, Fiscella K (2017). Who uses mobile phone health apps and does use matter? A secondary data analytics approach. J Med Internet Res.

[36] Yang E, Chang SJ, Ryu H, Kim HJ, Jang SJ (2020). Comparing factors associated with eHealth literacy between young and older adults. J Gerontol Nurs.

[37] Baumeister A, Aldin A, Chakraverty D, Monsef I, Tina J, Seven US, Görkem A, Kalbe E, Nicole S, Woopen C (2019). Interventions for improving health literacy in migrants. Cochrane Database Syst Rev.

[38] Heponiemi T, Jormanainen V, Leemann L, Manderbacka K, Aalto A, Hyppönen H (2020). Digital divide in perceived benefits of online health care and social welfare services: national cross-sectional survey study. J Med Internet Res.

[39] Zeiger W, DeBoer S, Probasco J (2020). Patterns and perceptions of smartphone use among academic neurologists in the United States: questionnaire survey. JMIR Mhealth Uhealth.

[40] Kirchberg J, Fritzmann J, Weitz J, Bork U (2020). eHealth literacy of German physicians in the pre-COVID-19 era: questionnaire study. JMIR Mhealth Uhealth.

[41] Cheng C, Beauchamp A, Elsworth GR, Osborne RH (2020). Applying the electronic health literacy lens: systematic review of electronic health interventions targeted at socially disadvantaged groups. J Med Internet Res.

[42] Peine A, Paffenholz P, Martin L, Dohmen S, Marx G, Loosen SH (2020). Telemedicine in Germany during the COVID-19 pandemic: multi-professional national survey. J Med Internet Res.

[43] Zimmermann BM, Fiske A, Prainsack B, Hangel N, McLennan S, Buyx A (2021). Early perceptions of COVID-19 contact tracing apps in German-speaking countries: comparative mixed methods study. J Med Internet Res.

[44] Menni C, Valdes AM, Freidin MB, Sudre CH, Nguyen LH, Drew DA, Ganesh S, Varsavsky T, Cardoso MJ, El-Sayed Moustafa JS, Visconti A, Hysi P, Bowyer RC, Mangino M, Falchi M, Wolf J, Ourselin S, Chan AT, Steves CJ, Spector TD (2020). Real-time tracking of self-reported symptoms to predict potential COVID-19. Nat Med.

[45] Boehm K, Ziewers S, Brandt MP, Sparwasser P, Haack M, Willems F, Thomas A, Dotzauer R, Höfner T, Tsaur I, Haferkamp A, Borgmann H (2020). Telemedicine online visits in urology during the COVID-19 pandemic-potential, risk factors, and patients' perspective. Eur Urol.

[46] Blustein DL, Guarino PA (2020). Work and unemployment in the time of COVID-19: the existential experience of loss and fear. J Humanistic Psychol.

[47] Hau H, Weitz J, Bork U (2020). Impact of the COVID-19 pandemic on student and resident teaching and training in surgical oncology. J Clin Med.

[48] Abel T, McQueen D (2020). Critical health literacy and the COVID-19 crisis. Health Promot Int.

[49] Schomakers E, Lidynia C, Vervier LS, Calero Valdez A, Ziefle M (2022). Applying an extended UTAUT2 model to explain user acceptance of lifestyle and therapy mobile health apps: survey study. JMIR Mhealth Uhealth.

[50] Seckin G, Hughes S (2021). Patient-reported outcomes in a nationally representative sample of older internet users: cross-sectional survey. JMIR Aging.

[51] De Santis KK, Jahnel T, Sina E, Wienert J, Zeeb H (2021). Digitization and health in Germany: cross-sectional nationwide survey. JMIR Public Health Surveill.

[52] Schaeffer D, Gille S (2021). Gesundheitskompetenz im Zeitalter der Digitalisierung. Präv Gesundheitsf.

[53] „Quelle: Internet“? Digitale Nachrichten- und Informationskompetenzen der deutschen Bevölkerung im Test. Studie März.

