# Supplementary material for: Use and Perception of Digital Health Technologies by Surgical Patients in Germany in the Pre–COVID-19 Era: Survey Study
Source: JMIR Form Res. 2022 May 20;6(5):e33985. doi: 10.2196/33985 (PMC9166644; doi:10.2196/33985)
Supplement: Multimedia Appendix 1 [file formative_v6i5e33985_app1.docx]

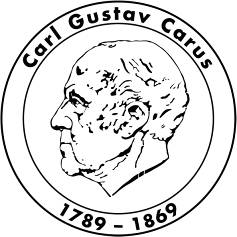
University Hospital Carl Gustav Carus

**Clinic and Polyclinic for Visceral, Thoracic and Vascular Surgery**

Clinic director: Prof. Dr. Jürgen Weitz

University Hospital Carl Gustav Carus - Fetscherstr. 74 - 01307 Dresden

Dear patient,

New digital information and communication technologies have changed many areas of our private and professional everyday life. Digital technologies, such as the Internet are also being used as a source of information in the health sector, and health apps are installed on smartphones.

At the same time, there are questions and uncertainties related to the use of these technologies and applications, in particular concerning the reliability of information, security and privacy aspects.

The purpose of this survey is to obtain an overview of the actual use by patients, as well as their knowledge and attitudes towards these new technologies and applications.

Answering the questions should not take more than 5-10 minutes. We would like to thank you very much for your help by filling in the anonymous questionnaire.

Your

Ulrich Bork

If you have any further questions about the project, I am happy to answer your questions.

PD Dr. Ulrich Bork, MBA

Head of the study centre

Clinic for Visceral, Thoracic and Vascular Surgery

University Hospital Dresden

***Information on participation in the study and data protection:***

*Your participation in this survey/study is voluntary. Whether or not you participate has no influence on your medical treatment.*

*By completing and submitting the questionnaire, you agree to participate in the study.*

*This survey is anonymous. This means that at no time will your personal data, such as name, date of birth, etc. be revealed. It is not possible to draw conclusions about individual persons, as no personal or related data is collected.*

*Data collected in the course of this study will be recorded and further evaluated anonymously.*

**1) General information:**

| **Your sex:** | **Male** | **Female** | **Divers** |
| --- | --- | --- | --- |

| **Your age (in years):** | | | | | | |
| --- | --- | --- | --- | --- | --- | --- |
| **≤ 30** | **31 - 40** | **41 - 50** | **51 - 60** | **61 - 70** | **71 - 80** | **≥ 81** |

| **How many inhabitants does your home town have?** | | | | |
| --- | --- | --- | --- | --- |
| **< 10.000** | **10.001 - 50.000** | **50.001 - 100.000** | **> 100.000** | **don’t know** |

| **How many different outpatient doctors do you go to regularly**  **(at least two a year) ?** (family doctor, dentist, gynaecologist, cardiologist, etc.) | | | | | |
| --- | --- | --- | --- | --- | --- |
| **None** | **Family doctor** | **2** | **3** | **4** | **>4** |

| **How often do you normally see a doctor per year?** (Outpatient and hospital) | | | | | |
| --- | --- | --- | --- | --- | --- |
| **Never** | **1x** | **2 - 3x** | **4-6x** | **7-9x** | **≥10x** |

| **What is your highest professional or academic degree?** | | |
| --- | --- | --- |
| **Still in training (**pupil, student, trainee) | |  |
| **School-leaving certificate** | |  |
| **Completed vocational training** | |  |
| **Completed academic degree (university, university of applied sciences)** | |  |
| **No school leaving qualification / no completed vocational training** | |  |
| **Not specified** | |  |
| **Do you suffer from a chronic disease?** (e.g. diabetes, high blood pressure, etc.) | | |
| **Yes** | **No** | |

**2) Questions about the use of the Internet**

| **Have you ever searched the Internet for diseases (e.g. via Google)?** | | |
| --- | --- | --- |
| **Yes** | **No** | **I don't know.** |

| **Before this (today's) stay in hospital, did you get information about your illness on the Internet?** | | |
| --- | --- | --- |
| **Yes** | **No** | |
| **How did you learn to use a computer and smartphone?** (multiple answers possible) | | |
| **Self-taught** | |  |
| **Internet research** | |  |
| **Family or friends** | |  |
| **Adult Education Centre** | |  |

| **Do you have a broadband Internet/DSL connection at home?** | | | |
| --- | --- | --- | --- |
| **No** | **Yes** | **I don't know.** |  |

**3) Questions about mobile phone (cell phone) and smartphone:**

| **Do you own a mobile phone?** | **Yes** | **No** |
| --- | --- | --- |

| **Is your mobile phone a smartphone?** | **I don't know.** | **Yes** | **No** |
| --- | --- | --- | --- |

| **What operating system does your smartphone use?** | | | |
| --- | --- | --- | --- |
| **iOS (Apple)** | **Android** | **Miscellaneous** | **Do not know/ Not applicable** |

| **Do you own and use a "smart fitness" or health product?** | |
| --- | --- |
| **No** |  |
| **Fitness bracelet/Smartwatch**  (e.g. Apple Watch, Samsung Galaxy Gear, Fibit, etc. ) |  |
| **Other "Connected Device"**  (e.g. body scale or blood pressure monitor with automatic storage on the smartphone) |  |

| **What do you use your smartphone/mobile phone for? (multiple answers possible):** | |
| --- | --- |
| **Telephone** |  |
| **Messenger / SMS** (e.g. Whatsapp, Threema, Telegram) |  |
| **Social media** (e.g. Facebook, Twitter, Instagram, Snapchat) |  |
| **Route planning / navigation** (e.g. maps, Google Maps, Tom Tom GO) |  |
| **Medical or health apps** |  |
| **Photography and photo use** |  |
| **listen to music** |  |
| **Watch movies / series** |  |
| **web browsing** |  |
| **Games** |  |
| **None / Not applicable** |  |
| **Miscellaneous:** |  |

| **Do you regularly use apps that affect your health?** | | |
| --- | --- | --- |
| **Yes** | **No** | **Not applicable/does not know** |

| **Which medical apps do you use or consider useful?** | **I find it useful** | **I use it** | **not useful** |
| --- | --- | --- | --- |
| **Medication App** (e.g. insulin schemes, medication reminder, etc.) |  |  |  |
| **Monitoring of vital signs** (e.g. monitoring of pulse, blood pressure, blood sugar, body temperature) |  |  |  |
| **Online appointment allocation / appointment coordination** |  |  |  |
| **App of the health insurance company with access to my patient data, findings, vaccination status, etc.** |  |  |  |
| **Fitness App for recording physical activity** |  |  |  |

**4) General questions on the use of digital technologies**

| **Do you think it would be useful to introduce online consultations (e.g. video consultation)?** *In a video consultation hour, patients can describe their complaints to the attending physician from home via the Internet using cameras and loudspeakers integrated in smartphones or computers, and a doctor-patient* conversation can be held *with the physician* without having to come to the practice. | | |
| --- | --- | --- |
| **Yes** | **No** | **I don't know.** |

| **Do you consider a digital patient file to be basically useful?** It gives outpatient doctors and hospitals access to your medical history, imaging findings such as X-rays, laboratory values, etc. etc., if you allow this. | | |
| --- | --- | --- |
| **Yes** | **No** | **I don't know.** |

| **Would you rather trust an app/a computer algorithm or a doctor connected via the Internet, during a video conference, to make a correct diagnosis?** | | | |  |
| --- | --- | --- | --- | --- |
| **App** | **real doctor (online)** | **None** | **I don't know.** | |

| **What disadvantages do you see in a video consultation with a telemedicine provider? (multiple answers possible)** | |
| --- | --- |
| **No disadvantages** |  |
| **Lack of personal contact** |  |
| **Doctor cannot examine me physically** |  |
| **Doctor is not known to me/anonymous** |  |
| **Lack of confidence in the competence of the doctor** |  |
| **No prescription of medication possible** |  |
| **Internet connection may not be secure against third parties** |  |

| **Would you take advantage of a video consultation in medical care?** | | | | |
| --- | --- | --- | --- | --- |
| **As often as possible** | | | |  |
| **Frequently** | | | |  |
| **Rather rare** | | | |  |
| **Not at all/almost not at all** | | | |  |
| **I don't know.** | | | |  |
| **Do you believe that the use of a fitness bracelet or a Smart Watch would improve or enhance your health?** | | | | |
| **Yes, very strong** | **Yeah, a little bit.** | **No** | **I don't know.** | |

**Your comments (free text):**
