# Supplementary material for: Use and Perception of Digital Health Technologies by Surgical Patients in Germany in the Pre–COVID-19 Era: Survey Study
Source: JMIR Form Res. 2022 May 20;6(5):e33985. doi: 10.2196/33985 (PMC9166644; doi:10.2196/33985)
Supplement: Multimedia Appendix 2 [file formative_v6i5e33985_app2.docx]

Supplementary Table 1. Participants’ demographic data by degree obtained. Given are total n for each question as well as frequency (n, %). A total of 406 questionnaires were submitted and evaluated, 370 participants provided information about their degree.

| Characteristic | | Academic degree | Other degree /no degree | Total |
| --- | --- | --- | --- | --- |
| N | | 123 | 247 | 370 |
| **Gender, n (%)** | | | | |
|  | Male | 80 (65.6) | 128 (52.2) | 208 (56.7) |
|  | Female | 42 (34.4) | 117 (47.8) | 159 (43.3) |
|  | *Total* | *122 (99.2)* | *245 (99.2)* | *367* |
| **Age group, n (%)** | | | | |
|  | 18 - 40 y | 16 (13.0) | 44 (17.9) | 60 (16.3) |
|  | 41 – 70 y | 76 (61.8) | 162 (65.9) | 238 (64.5) |
|  | ≥ 71 y | 31 (25.2) | 40 (16.3) | 71 (19.2) |
|  | *Total* | *123 (100)* | *246 (99.6)* | *369* |
| **No. inhabitants home town, n (%)** | | | | |
|  | < 10k | 44 (36.4) | 78 (33.2) | 122 (34.3) |
|  | 10-50k | 26 (21.5) | 64 (27.2) | 90 (25.3) |
|  | 50-100k | 2 (1.7) | 14 (6.0) | 16 (4.5) |
|  | >100k | 46 (38.0) | 53 (22.6) | 99 (27.8) |
|  | Not known | 3 (2.5) | 26 (11.1) | 29 (8.1) |
|  | *Total* | *121 (98.4)* | *235 (95.1)* | *356* |
| **Regularly visited physicians, n (%) ^a^** | | | | |
|  | None | 5 (4.1) | 17 (6.9) | 22 (5.9) |
|  | Family doctor | 109 (88.6) | 212 (85.8) | 321 (86.8) |
|  | Cardiologist | 22 (17.9) | 19 (7.7) | 41 (11.1) |
|  | Gastroenterologist | 11 (8.9) | 23 (9.3) | 34 (9.2) |
|  | Other doctor | 48 (39.0) | 82 (33.2) | 130 (35.1) |
|  | *Total* | *123 (100)* | *247 (100)* | *370* |
| **No. outpatient and hospital visits per year, n (%)** | | | | |
|  | Never | 1 (0.8) | 4 (1.6) | 5 (1.4) |
|  | 1x | 10 (8.3) | 19 (7.8) | 29 (8.0) |
|  | 2-3x | 43 (35.8) | 78 (32.0) | 121 (33.2) |
|  | 4-6x | 41 (34.2) | 74 (30.3) | 115 (31.6) |
|  | 7-9x | 8 (6.7) | 35 (14.3) | 43 (11.8) |
|  | ≥10x | 17 (14.2) | 34 (13.9) | 51 (14.0) |
|  | *Total* | *120 (97.6)* | *244 (98.8)* | *364* |
| **Chronic disease, n (%)** | | | | |
|  | Yes | 77 (63.1) | 129 (54.4) | 206 (57.4) |
|  | No | 45 (36.9) | 108 (45.6) | 153 (42.6) |
|  | *Total* | *122 (99.2)* | *237 (96.0)* | *359* |

^a^ Multiple answers were possible, the percentage refers to the respective total.

Supplementary Table 2. Internet usage by participants sorted by obtained degree. Given are total n for each question as well as frequencies (n, %).

| Question | | Academic degree | Other | Total |
| --- | --- | --- | --- | --- |
| N | | 123 | 247 |  |
| **Searching the internet for diseases, n (%)** | | | | |
|  | Yes | 97 (79.5) | 160 (65.6) | 257 (70.2) |
|  | No | 25 (20.5) | 82 (33.6) | 107 (29.2) |
|  | Not known | 0 | 2 (0.8) | 2 (0.5) |
|  | *Total* | *122 (99.2)* | *244 (98.8)* | *366* |
| **Searching online information about current illness, n (%)** | | | | |
|  | Yes | 73 (60.3) | 100 (41.7) | 173 (47.9) |
|  | No | 48 (39.7) | 140 (56.7) | 188 (52.1) |
|  | *Total* | *121 (98.4)* | *240 (97.2)* | *361* |
| **Learning to use a computer / smartphone, n (%) ^a^** | | | | |
|  | Self-taught | 86 (69.9) | 154 (62.3) | 240 (64.9) |
|  | Internet research | 12 (9.8) | 13 (5.3) | 25 (6.8) |
|  | Family / friends | 41 (33.3) | 103 (41.7) | 144 (38.9) |
|  | Adult education center | 5 (4.1) | 4 (1.6) | 9 (2.4) |
|  | Other | 13 (10.6) | 15 (6.1) | 28 (7.6) |
|  | *Total* | *123 (100)* | *247 (100)* | *370* |
| **DSL / broadband connection at home, n (%)** | | | | |
|  | Yes | 101 (83.5) | 159 (65.4) | 260 (71.4) |
|  | No | 17 (14.0) | 64 (26.3) | 81 (22.3) |
|  | Not known | 3 (2.5) | 20 (8.2) | 23 (6.3) |
|  | *Total* | *121 (98.4)* | *243 (98.4)* | *364* |

^a^ Multiple answers were possible, the percentage refers to the respective total.

Supplementary Table 3. Questions about the use of mobile phones / cell phones /smart phones by participants sorted by obtained degree. Given are total n for each question as well as frequencies (n, %).

|  | | Academic degree | Other | Total |
| --- | --- | --- | --- | --- |
|  | | 123 | 247 | 370 |
| **Owning a mobile phone, n (%)** | | | | |
|  | Yes | 118 (95.9) | 227 (93.4) | 345 (94.3) |
|  | No | 5 (4.1) | 16 (6.6) | 21 (5.7) |
|  | *Total* | *123 (100)* | *243 (98.4)* | *366* |
| **Mobile phone is a smartphone, n (%)** | | | | |
|  | Yes | 101 (84.9) | 184 (77.3) | 285 (79.8) |
|  | No | 16 (13.4) | 50 (21.0) | 66 (18.5) |
|  | Not known | 2 (1.7) | 4 (1.7) | 6 (1.7) |
|  | *Total* | *119 (96.8)* | *238 (96.4)* | *357* |
| **Operating system of smartphone, n (%)** | | | | |
|  | iOS | 34 (31.5) | 42 (20.4) | 76 (24.2) |
|  | Android | 59 (54.6) | 123 (59.7) | 182 (58.0) |
|  | Misc | 7 (6.5) | 13 (6.3) | 20 (6.4) |
|  | Not known | 8 (7.4) | 28 (13.6) | 36 (11.5) |
|  | *Total* | *108 (87.8)* | *206 (83.4)* | *314* |
| **Owning a fitness device, n (%)** | | | | |
|  | No | 98 (81.7) | 182 (81.6) | 280 (81.6) |
|  | Fitness bracelet / smartwatch | 21 (17.5) | 31 (13.9) | 52 (15.2) |
|  | Yes, other | 1 (0.8) | 10 (4.5) | 11 (3.2) |
|  | *Total* | *120 (97.6)* | *223 (90.3)* | *343* |
| **Using smartphone / mobile phone for… , n (%) ^a^** | | | | |
|  | Phone calls | 113 (91.9) | 202 (81.8) | 315 (85.1) |
|  | Messenger services / SMS | 89 (72.4) | 155 (62.8) | 244 (65.9) |
|  | Social media | 33 (26.8) | 70 (28.3) | 103 (27.8) |
|  | Route planning / navigation | 69 (56.1) | 115 (46.6) | 184 (49.7) |
|  | Medical or health apps | 24 (19.5) | 32 (13.0) | 56 (15.1) |
|  | Photography and photo use | 82 (66.7) | 155 (62.8) | 237 (64.1) |
|  | Listening to music | 32 (26.0) | 71 (28.7) | 103 (27.8) |
|  | Watching movies / series | 13 (10.6) | 37 (15.0) | 50 (13.5) |
|  | Web browsing | 69 (56.1) | 113 (45.7) | 182 (49.2) |
|  | Games | 18 (14.6) | 50 (20.2) | 68 (18.4) |
|  | None / not applicable | 0 | 2 (0.8) | 2 (0.5) |
|  | Other | 3 (2.4) | 3 (1.2) | 6 (1.6) |
|  | *Total* | *123 (100)* | *247 (100)* | *370* |
| **Using apps affecting health, n (%)** | | | | |
|  | Yes | 19 (16.0) | 22 (9.7) | 41 (11.9) |
|  | No | 96 (80.7) | 201 (88.9) | 297 (86.1) |
|  | Not known / not applicable | 4 (3.4) | 3 (1.3) | 7 (2.0) |
|  | *Total* | *119 (96.7)* | *226 (91.5)* | *345* |

^a^ Multiple answers were possible, the percentage refers to the respective total.

Supplementary Table 4. General questions about the use of digital technologies by participants sorted by obtained degree. Given are total n for each question as well as frequencies (n, %). .

| Questions | | Academic degree | Other | Total |
| --- | --- | --- | --- | --- |
|  | | 123 | 247 | 370 |
| **Do you think it would be useful to introduce online consultations?, n (%)** | | | | |
|  | Yes | 47 (38.5) | 63 (27.5) | 110 (31.3) |
|  | No | 50 (41.0) | 100 (43.7) | 150 (42.7) |
|  | Not known | 25 (20.5) | 66 (28.8) | 91 (25.9) |
|  | *Total* | *122 (99.2)* | *229 (92.7)* | *351* |
| **Do you consider an electronic health record to be basically useful?, n (%)** | | | | |
|  | Yes | 101 (84.2) | 161 (70.6) | 262 (75.3) |
|  | No | 6 (5.0) | 31 (13.6) | 37 (10.6) |
|  | Not known | 13 (10.8) | 36 (15.8) | 49 (14.1) |
|  | *Total* | *120 (97.6)* | *228 (92.3)* | *348* |
| **Do you trust in … for making a correct diagnosis?, n (%)** | | | | |
|  | App | 4 (3.4) | 2 (0.9) | 6 (1.8) |
|  | Real doctor (online) | 70 (59.3) | 96 (45.1) | 166 (50.2) |
|  | None | 24 (20.3) | 52 (24.4) | 76 (23.0) |
|  | Not known | 20 (16.9) | 63 (29.6) | 83 (25.1) |
|  | *Total* | *118 (95.9)* | *213 (86.2)* | *331* |
| **Do you see disadvantages in a video consultation with a telemedicine provider?, n (%) ^a^** | | | | |
|  | No disadvantages | 6 (4.9) | 12 (4.9) | 18 (4.9) |
|  | Lack of personal contact | 79 (64.2) | 134 (54.3) | 213 (57.6) |
|  | No physical examination | 96 (78.0) | 156 (63.2) | 252 (68.1) |
|  | Doctor unknown / anonymous | 44 (35.8) | 81 (32.8) | 125 (33.8) |
|  | Lack of confidence in competence of the doctor | 28 (22.8) | 73 (29.6) | 101 (27.3) |
|  | No prescription of medication possible | 54 (43.9) | 80 (32.4) | 134 (36.2) |
|  | Unsecure internet connection | 53 (43.1) | 93 (37.7) | 146 (39.5) |
|  | Other | 0 | 4 (1.6) | 4 (1.1) |
|  | *Total* | *123 (100)* | *247 (100)* | *370* |
| **Would you take advantage of a video consultation in medical care?, n (%)** | | | | |
|  | As often as possible | 8 (7.0) | 9 (4.3) | 17 (5.2) |
|  | Frequently | 20 (17.4) | 18 (8.6) | 38 (11.7) |
|  | Rather rarely | 34 (29.6) | 58 (27.6) | 92 (28.3) |
|  | (Almost) Not at all | 35 (30.4) | 90 (42.9) | 125 (38.5) |
|  | Not known | 18 (15.7) | 35 (16.7) | 53 (16.3) |
|  | *Total* | *115 (93.5)* | *210 (85.0)* | *325* |
| **Would the use of a fitness bracelet or a Smart Watch improve or enhance your health?, n (%)** | | | | |
|  | Yes, very strong | 7 (6.0) | 10 (4.6) | 17 (5.1) |
|  | Yeah, a little bit | 24 (20.7) | 40 (18.3) | 64 (19.2) |
|  | No | 59 (50.9) | 109 (50.0) | 168 (50.3) |
|  | Not known | 26 (22.4) | 59 (27.1) | 85 (25.4) |
|  | *Total* | *116 (94.3)* | *218 (88.3)* | *334* |

^a^ Multiple answers were possible, the percentage refers to the respective total.

Supplementary Table 5. Use and usefulness of medical apps as assessed by participating participants sorted by obtained degree. Given are total n for each question as well as frequencies (n, %).

| Apps | | Academic degree | Other | Total |
| --- | --- | --- | --- | --- |
|  | | 123 | 247 | 370 |
| **Medication app (e.g. reminder, insulin scheme, etc.), n (%)** | | | | |
|  | Finding it useful | 39 (53.4) | 68 (59.6) | 107 (57.2) |
|  | Using it | 11 (15.1) | 11 (9.6) | 22 (11.8) |
|  | Not useful | 23 (31.5) | 35 (30.7) | 58 (31.0) |
|  | *Total* | *73 (59.3)* | *114 (46.2)* | *187* |
| **Monitoring of vital signs (e.g. pulse, blood sugar, etc.), n (%)** | | | | |
|  | Finding it useful | 34 (44.2) | 59 (52.2) | 93 (48.9) |
|  | Using it | 15 (19.5) | 12 (10.6) | 27 (14.2) |
|  | Not useful | 28 (36.4) | 42 (37.2) | 70 (36.8) |
|  | *Total* | *77 (62.6)* | *113 (45.7)* | *190* |
| **Online appointment allocation / coordination, n (%)** | | | | |
|  | Finding it useful | 47 (65.3) | 63 (57.3) | 110 (60.4) |
|  | Using it | 15 (20.8) | 14 (12.7) | 29 (15.9) |
|  | Not useful | 10(13.9) | 33 (30.0) | 43 (23.6) |
|  | *Total* | *72 (58.5)* | *110 (44.5)* | *182* |
| **App of the health insurance company with access to my patient data, findings, vaccination status, etc., n (%)** | | | | |
|  | Finding it useful | 36 (50.7) | 53 (49.5) | 89 (50.0) |
|  | Using it | 13 (18.3) | 9 (8.4) | 22 (12.4) |
|  | Not useful | 22 (31.0) | 45 (42.1) | 67 (37.6) |
|  | Total | *71 (57.7)* | *107 (43.3)* | *178* |
| **Fitness App for recording physical activity, n (%)** | | | | |
|  | Finding it useful | 32 (41.6) | 43 (39.1) | 75 (40.1) |
|  | Using it | 18 (23.4) | 17 (15.5) | 35 (18.7) |
|  | Not useful | 27 (35.1) | 50 (45.5) | 77 (41.2) |
|  | Total | *77 (62.6)* | *110 (44.5)* | *187* |
